# High dynamic range scanning tunneling microscopy

**DOI:** 10.1016/j.mex.2024.102857

**Published:** 2024-07-10

**Authors:** Ajla Karić, Carolina A Marques, Berk Zengin, Fabian Donat Natterer

**Affiliations:** Department of Physics, University of Zurich, Winterthurerstrasse 190, Zurich CH-8057, Switzerland

**Keywords:** Scanning tunneling microscopy, Scanning tunneling spectroscopy, Dynamic range, Active power Filter, Amplifier saturation, Band-structure, Density of states, High Dynamic Range Scanning Tunneling Microscopy

## Abstract

We increase the dynamical range of a scanning tunneling microscope (STM) by actively subtracting dominant current-harmonics generated by nonlinearities in the current-voltage characteristics that could saturate the current preamplifier at low junction impedances or high gains. The strict phase relationship between a cosinusoidal excitation voltage and the current-harmonics allows excellent cancellation using the displacement-current of a driven compensating capacitor placed at the input of the preamplifier. Removal of DC currents has no effect on, and removal of the first harmonic only leads to a rigid shift in differential conductance that can be numerically reversed by adding the known removal current. Our method requires no permanent change of the hardware but only two phase synchronized voltage sources and a multi-frequency lock-in amplifier to enable high dynamic range spectroscopy and imaging.

• Active power filter

• Dynamic range compression

• High gain preamplifier

## Graphical abstract

**Figure:**
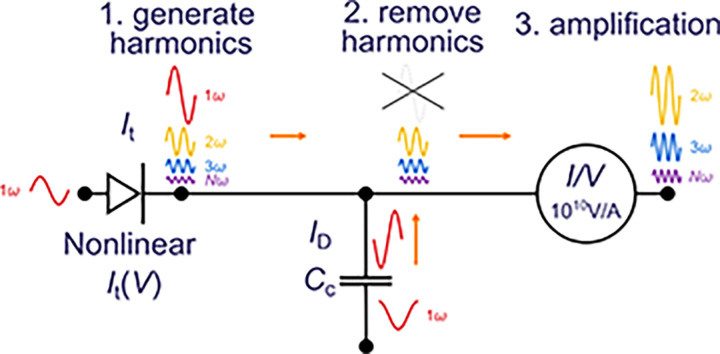

Specifications table

**Table:** 

Subject area:	*Physics and Astronomy*
More specific subject area:	*Condensed Matter Physics*
Name of your method:	*High Dynamic Range Scanning Tunneling Microscopy*
Name and reference of original method:	*NA*
Resource availability:	*NA*

## Background

The hallmark of the scanning tunneling microscope (STM) is the exponential current-distance relationship demonstrated by Binnig and coworkers in 1982 [1], just before wowing the world with their atomic resolution imaging of the 7 × 7 reconstruction of the silicon surface [2]. These early milestones established the need for current preamplifiers that would span a large dynamical range. Even at constant tip-sample separation, tunneling spectroscopy quickly runs into preamplifier saturation problems when trying to simultaneously measure electronic states close to and far away from the Fermi level. This is unsatisfactory when one is interested in the local density of states, whose measurement throws out the large current background in the current derivative d*I*_t_(*V*)/d*V*. Large bias range spectroscopy then frequently requires a lower preamplifier gain that consequently limits the minimally measurable current and thus obscures information around the Fermi level. Other STM pioneers cleverly circumvented the dynamical range limitation by synchronizing the lowering of the bias voltage with the closing of the tip-sample separation [3], which however, required an a*-posteriori* correction using the separately measured global decay length.

Here we introduce a high-dynamical range STM (HDR-STM) concept that actively compresses the dynamical range of the current, allowing us to use higher gains at small junction-impedances without saturating the preamplifier. The large gain enables us to resolve the small current amplitudes that would be lost in low gain measurements. Our approach requires no assumptions of the tunneling junction or spectroscopically synchronized tip-motion and it relies solely on the strict phase relationship between a cosinusoidal excitation voltage and the current-harmonics naturally generated by the nonlinear current-voltage characteristics *I*_t_(*V*). Our method is conceptually similar to capacitive displacement current compensation, but we inject displacement currents to principally remove resistive as well as capacitive currents. It can also be interpreted as a complementary active power filter that removes low-order instead of high-order harmonics.

### Method details

**Fig. 1:**
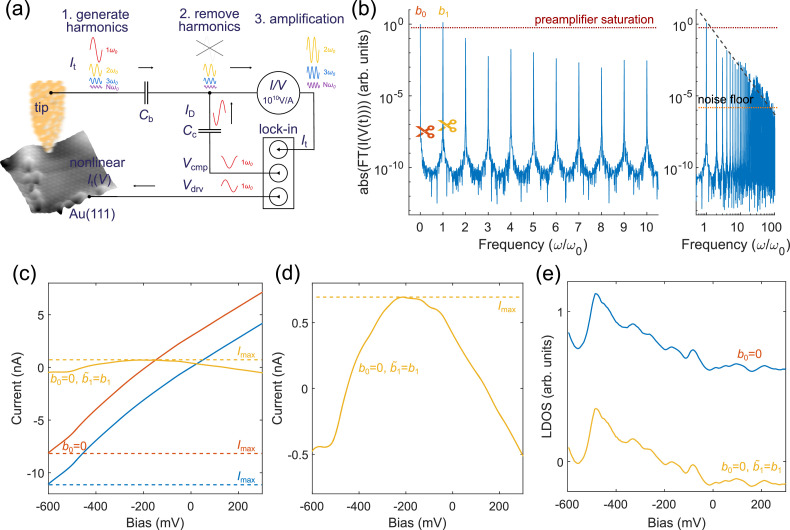
High dynamic range scanning tunneling microscopy concept. (a) The application of a harmonic excitation *V*(*t*)=*V*_drv_ cos(*ω*_0_*t*) on the nonlinearities *I*_t_(V) of the tunneling junction creates higher order current harmonics that can be subtracted by a tailored displacement current *I*_D_ created with the compensating capacitor *C*_c_ and the application of *V*_cmp_. The optional DC blocking capacitor *C*_b_ removes the DC current component (*b*_0_) to further increase the dynamical range of the STM. The atomically resolved Au(111) surface was measured without DC (*b*_0_) and first harmonic current (*b*_1_) at preamplifier gain of 10^10^ at an effective current of about 50 nA (*V*_drv_=150 mV, *f* = 640 Hz, *b̃*_1_≈50 nA, *T* = 4.3 K). (b) Simulated harmonic decomposition of *I*_t_(*V*) of Au(111) after excitation with *V*(*t*)=*V*_drv_ cos(*ω*_0_*t*) using a conventionally measured spectrum. The removal of high amplitude current harmonics (scissor symbols) prevents preamplifier saturation (dotted red line). The panel on the right shows the harmonic decomposition up to higher order harmonics, where the dashed dark-gray line shows the 1/ω trend of the higher order harmonics and the dotted orange line indicates the noise level of the current preamplifier. A smaller tip-sample distance would lift more harmonics above the noise level. (c) *I*_t_(*V*) curves created using all harmonics (blue), without DC component (*b*_0_, red), and without DC (*b*_0_) and first harmonic (*b*_1_, yellow). The latter shows the strongest compression of the current range, enabling the use of larger preamplifier gains. (d) Zoom into compressed current from (c). (e) The removal of the DC component (*b*_0_) has no effect on the local density of states (LDOS ∝ d*I*_t_(*V*)/d*V*, blue), and the removal of the first harmonic only rigidly shifts the LDOS (yellow). The original *I*_t_(*V*) and LDOS could be reconstructed using the known amplitude of *b*_1_ while the removal of the DC component (*b*_0_) only impacts *I*_t_(*V*), albeit irreversibly.

The application of a cosinusoidal voltage *V*(*t*)=*V*_drv_cos(*ω*_0_*t*) on the nonlinear current-voltage characteristics *I*_t_(*V*) of a tunneling junction generates a time-varying tunneling current whose frequency content is composed of integer order harmonics *n*ω_0_ of the excitation frequency *ω*_0_ [4,5] (see concept in Fig. 1). We write *I*_t_(*V*) as an infinite sum of increasing order polynomials, and apply *V*(*t*) to write $I_{t}\left(V(t)\right)=a_{0}+\sum_{n=1}^{\infty }a_{n}\text{co}s^{n}\left(\omega_{0}t\right)=b_{0}+\sum_{n=1}^{\infty }b_{n}\text{cos}\left(n\omega_{0}t\right)$, where we used trigonometric identities^2^ to see how the *n*^th^ power transforms the excitation voltage into an *n*^th^-order current-harmonic. This harmonic decomposition can be simulated using a pre-measured *I*_t_(*V*) curve, shown in Fig. 1(b) for an *I*_t_(*V*) of Au(111). While the *b*_0_ coefficient contains contributions from all even order polynomials, the *b*_n>1_ coefficients carry contributions from polynomials smaller or equal to *n*, and they exhibit the characteristic 1/$n\omega_{0}$ trend [see dark gray dashed line in Fig. 1(b) right panel]. The transformation of the tunneling current into harmonics provides the basis of how their measurement can be used to reconstruct the original *I*_t_(*V*) characteristics [4,5]. Note that the maximally possible tunneling current of this harmonic decomposition is $I_{t}^{max}=\sum_{n=0}^{\infty }\left|b_{n}\right|$, which is dominated by the lowest integer components, *b*_0_ and *b*_1_. While the strong attenuation of *b*_n_ of the higher order harmonics allows the faithful reconstruction of the original *I*_t_(*V*) curve by only measuring a finite number of the current harmonics, the large amplitudes of the DC component *b*_0_ and low integer harmonics may saturate the preamplifier, requiring the use of lower gains. Unfortunately, the reduced preamplifier gain then raises the noise floor and increases the minimally detectable current. The larger noise floor also means that higher order harmonics would become obscured by noise, which would lead to a loss of information.

Fortunately, the transformation of the tunneling current into harmonics allows for the straightforward implementation of an active power filter that removes current harmonics by exploiting the perfect and stable phase synchronization between the dominant (or any desired) harmonic and the cosinusoidal excitation voltage. This works by applying a synchronized voltage *V*(*t*)=*V*_cmp_cos(*nω*_0_*t+φ*), with *φ≈*π/2 and *n* designating the targeted harmonic, onto a compensating capacitor *C*_c_ placed at the input of the preamplifier. This capacitor then acts as a current source that pushes a displacement current *I*_D_=*C*_c_d*V*/d*t=b̃*_n_ onto the current line, perfectly opposing the respective current *before* reaching the preamplifier [Fig. 1(a)]. Principally, this allows compressing the maximal current down to arbitrarily small values $I_{t}^{HDR}=I_{t}^{max}-\sum_{n=1}^{N}\left|{\tilde{b}}_{n}\right|$ by removing any selection of the current harmonics, [Fig. 1(b)], simply by applying a superposition of properly phase shifted and scaled integer harmonics of the excitation voltage on *C*_c_. This harmonic removal then enables operating the preamplifier at the maximal possible gain to substantially increase the dynamical range and signal-to-noise ratio of the STM. The example of Fig. 1(c) shows the reduction of the maximal current (*I*_max_) amplitudes during spectroscopy when the DC component (*b*_0_) or *b*_1_ are removed [see also zoom in Fig. 1(d)]. Since *I*_max_ determines the saturation of the preamplifier, it becomes immediately clear that the compressed spectrum could now be measured at a higher gain.

At first sight, the removal of current harmonics might appear unacceptable because it seemingly discards information about the tunneling junction, but we recall that we have perfect knowledge of these missing currents as they are equivalent to their compensating displacement currents. Information is irreversibly lost, however, if one removed the zero-frequency component *b*_0_, for instance by using the (optional) DC blocking capacitor *C*_b_ at the preamplifier input. The latter might be acceptable, if the researcher was mostly interested in differential conductance rather than absolute current values, for instance when focusing on d*I*_t_(*V*)/d*V*, such as for quasiparticle interference measurements [6]. The effect of *b*_0_ and *b*_1_ removal on the local density of states measured via d*I*_t_(*V*)/d*V* can be seen in Fig. 1(e). Removing *b*_1_ only leads to a rigid shift towards negative LDOS while preserving the overall shape of the spectrum.

### Method validation

We are now looking at the experimental implementation of our active power filter concept for HDR-STM. Our method requires only the reversible addition of the compensating capacitor *C*_c_ (1 pF) placed via a tee-piece at the input of the preamplifier and the optional series DC blocking capacitor *C*_b_ (100 pF) that removes the *b*_0_ component of the current [Fig. 1(a)]. The 1 pF value was chosen because it matches the effective stray capacitance of our junction and the 100 pF provides a low impedance for the resistive current harmonics across the blocking capacitor. These capacitance values could be good starting points for initial tests and may vary in other systems or after further optimization. To generate and compensate the current harmonics *b*_n_, we use a synchronized dual channel waveform generator, implemented in a multifrequency lock-in amplifier (Intermodulation products, MLA-3) whose first output acts as the excitation voltage *V*(*t*)=*V*_drv_cos(*ω*_0_*t*) and the second output *V*(*t*)=*V*_cmp_cos(*ω*_0_*t+φ*) in combination with *C*_c_ as the harmonic-cancelling current source. The tunneling current is fed into a large bandwidth preamplifier (NF corp SA-606F2: gain 10^9^ V/A or NF corp SA-607F2: gain 10^10^ V/A), whose signals provide current information for STM operation and for harmonic demodulation at the lock-in amplifier. The preamplifier bandwidth determines the frequency of the highest harmonic that may still reach the lock-in amplifier, which in our case is 40′320 Hz for the 63^rd^ harmonic of our preferred excitation frequency of *f*=640 Hz.

We have previously used the compensating capacitor *C*_c_ to accurately cancel out the stray displacement current *I*_D_ using *V*(*t*)=*V*_drv_*C_c_*/*C_s_*cos(*ω*_0_*t+φ*_c_), with *φ_c_*≈π [5] to produce an opposing current. In addition to that stray capacitive current compensation, we now also apply *V*_cmp_=*V*_cmp_cos(*ω_0_t+φ*), *φ*=*φ_c_*-π/2 onto *C*_c_ to deliberately generate *b̃*_n_=*C*_c_d*V*(*t*)/d*t* that eliminates the strong resistive harmonics generated by the *I*_t_(*V*) curve. Since capacitive and resistive currents have a π/2 phase shift, we first compensate the capacitive displacements currents due to stray capacitances to fix the phase relationship. Since the capacitor is linear within the parameters used in our method, the second output can be set up to create the superposition of the individual removal currents for all harmonics. Our setup requires no modification other than the application of suitably large cancellation voltages or adjustments to the compensating capacitance *C*_c_. Our presently used *C*_c_=1 pF and the 12 V maximal excitation amplitude, allows current compensation up to about 50 nA. Accordingly, larger currents of small junction impedances could be compensated by increasing *C*_c_. Note that we also compensate for the frequency dependent transfer function of amplitudes and phases for all harmonic frequencies, as described previously [5,7]. The measurement of the transfer function is carried out with the tip retracted by a few nanometers, in a routine that also deals with the cancellation of the stray capacitance related displacement current. We obtain the amplitude transfer function by applying the same AC amplitude for all harmonic frequencies and measure how the displacement current deviates from the expected *I*_D_= *C*_s_*nω*_0_*V*. At the same time, we also measure the phase shift of the frequencies *n*ω_0_ relative to *ω_0_*.

**Fig. 2:**
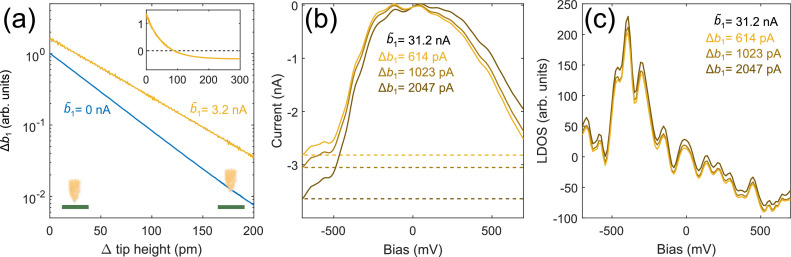
High dynamic range spectroscopy. (a) Tip height dependence of the difference (Δ*b*_1_) between the amplitude of the first harmonic and the current from active compensation. Without harmonic removal (*b̃*_1_=0 nA), the amplitude of the first harmonic follows an exponential trend (blue, *b*_0_=0, *V*_drv_=600 mV, *f* = 640 Hz, gain 10^9^ V/A, *T* = 4.3 K). With harmonic compensation active (*b̃*_1_=3.2 nA), we numerically subtract an offset corresponding to the out-of-tunneling displacement current to emulate a zero current situation for large tip-sample separations (the inset shows Δ*b*_1_ before offset subtraction, zero corresponds to the tip-height where the harmonic removal current exactly matches the resistive current). The height-dependence in tunneling then also follows an exponential trend (yellow, *b*_0_≠0, *V*_drv_=700 mV, *f* = 640 Hz, gain 10^9^ V/A, *T* = 1.4 K). The distinct slopes are attributed to the different junction work-functions of the two experimental runs. (b) I_t_(V) spectroscopy of Au(111) with active harmonic removal of *b̃*_1_=31.2 nA at different tip-heights. At small Δ*b*_1_=*b*_1_-*b̃*_1_, the current-range is more compressed and the resistive part better matches the active compensation current b̃_1_ (b̃_1_=31.2 nA, *V*_drv_=600 mV, *f* = 640 Hz, gain 10^9^ V/A, *T* = 4.3 K). The DC component was not removed (*b*_0_≠0) and the dashed lines mark the respective maximal currents I_max_. (c) d*I*_t_(*V*)/d*V* spectra with active compensation, numerically differentiated from (b). The LDOS shifts rigidly towards negative values, the better we remove *b*_1_ using active compensation. Importantly, the harmonic removal preserves the spectral structures, as can be seen by the coincidence of the LDOS features between the three different traces.

We next validate our HDR-STM method for tunneling spectroscopy, notably to verify the benign action of the harmonic removal on local density of states (LDOS) measurements. Using *V*_drv_=600 mV, we start by examining the height-dependence of the harmonic removal, characterized by Δ*b*_1_=*b*_1_-*b̃*_1_ as shown in Fig. 2(a). The blue line shows Δ*b*_1_ without active compensation (*b̃*_1_=0) as it follows the same exponential distance-dependence that is found for the tunneling current. This exponential trend allows *b*_1_ to be used for feedback control of the tip-height [8], which may be useful to safely scan the surface even without DC current or when the time-averaged current would be zero for nearly ohmic or electron-hole symmetric junctions. When we apply a fixed removal current *b̃*_1_ via *C*_c_ while lifting the tip out-of-contact, we measure the capacitive contribution to the displacement current Δ*b*_1_ in the lock-in amplifier that first shrinks towards zero when we set the tip-sample separation to the exact height where *b*_1_ exactly matches *b̃*_1_ [see inset in Fig. 2(a)]. When we move the tip even closer to the surface, the resistive *b*_1_ current is larger than the harmonic removal current *b̃*_1_ and we observe a sign change in Δ*b*_1_. In order to use Δ*b*_1_ as feedback signal, we numerically subtract an offset corresponding to the out-of-contact displacement current such that Δ*b*_1_ becomes zero at large tip-sample distances and it then only grows exponentially (yellow) when the tip is moved closer to the surface, making it suitable for feedback control, as before.

Fig. 2(b) shows the reconstructed *I*_t_(*V*) curves measured on Au(111) with varying magnitude of *b*_1_ removed using a compensation current of *b̃*_1_=31.2 nA, which would saturate the 10^9^ gain of the preamplifier. The current with active harmonic removal shows the same shape as found in the simulation of Fig. 1(d). The closer the resistive (*b*_n_) and removal currents (*b̃*_n_) match (Δ*b*_1_→0), the smaller is *I*_max_, and the more compressed is the spectrum. The crossing point of the three curves corresponds to the zero-bias condition and it can accordingly be used to quantify bias-offsets present in the system. If we additionally removed *b*_0_, the compressed *I*_t_(V) curve would center around the zero current mark, further reducing *I*_max_, as also seen in Fig. 1(d). The LDOS in Fig. 2(c) clearly shows the onset of the characteristic Au(111) surface state at about −500 mV as it does for conventionally measured spectra using much smaller effective currents. The removal of *b*_1_ only rigidly shifts the LDOS but preserves the spectral details, as can be seen from the coinciding wiggles among the three d*I*_t_(*V*)/d*V* traces.

**Fig. 3:**
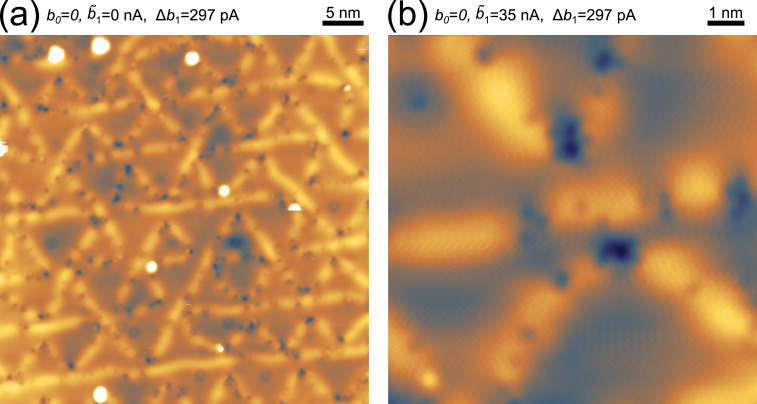
Topographic measurement using HDR-STM. (a) Topography of Au(111) without DC current (*b*_0_=0) and feedback loop on log(Δ*b*_1_) (*V*_drv_=600 mV, Δ*b*_1_=297 pA, *f*=640 Hz, gain 10^9^ V/A, *T*=4.3 K). The triangular pattern is the impurity pinned herringbone reconstruction. (b) Closed loop topography to maintain Δ*b*_1_=297 pA, while actively subtracting *b̃*_1_=36.45 nA (*b*_0_=0, *V*_drv_=600 mV, *f*=640 Hz, gain 10^9^ V/A, *T*=4.3 K).

Fig. 3(a) shows a topographic image of Au(111), measured with a *C*_b_=100 pF DC blocking capacitor (*b*_0_=0), *V*_drv_=600 mV and a feedback loop maintaining constant log(Δ*b*_1_). In this mode, there is no more galvanic connection between the tip and the preamplifier circuit. Fig. 3(b) shows a topographic scan while actively removing a large part of the resistive current carried by the first harmonic. This scan operates by setting the feedback controller to maintain log(Δ*b*_1_)=300 pA while having *b̃*_1_=36 nA active. We have also achieved atomic resolution in HDR-STM mode, as shown in Fig. 1(a), which we measured at *b̃*_1_≈50 nA and at a gain of 10^10^ or equivalent to a dynamical range of 146 dB^3^. Our specified current estimate is here approximate because the gain limited the maximal current to 1 nA, which was occasionally exceeded with the second order harmonic.

## Limitations

When implementing the high dynamic range STM method, we encountered a few limitations that we discuss in the following.

The first is the large value of the DC component *b*_0_ that can grow to the same order of magnitude than the first harmonic [see simulation in Fig. 1(b)], because all even order polynomials contribute to *b*_0_ via the polynomial decomposition mentioned above. The large value of *b*_0_ has motivated our use of the blocking capacitor *C*_b_ in parts of the experimental verification, despite its irreversibility on the current reconstruction. For future studies related to quasiparticle interference, we may be able to accept this loss of information because those studies will mostly focus on the spatial dependence of d*I*_t_(*V*)/d*V*. One remedy for a large *b*_0_ could be to add a DC voltage *V*(*t*)=*V*_DC_+*V*_drv_ cos(*ω*_0_*t*) that maximizes the amplitude of odd order harmonics, which can be simulated using conventionally measured spectra.

A second limitation is the cancellation of the current only after transfer across the tunneling junction. This may lead to noticeable Joule heating in extreme cases. For instance, the impedances used by Binnig [1] and Limot [9], would dissipate a power equivalent of order 1 µW. The Feenstra spectroscopy method [10] would then be a suitable alternative but its normalization protocol requires the measurement of the decay length for every spectroscopy location and the consideration of potential tip-gating effects [11]. On the other hand, the Feenstra normalization protocol (d*I*_t_/d*V)*/(*I*_t_/*V)* appears compatible with our HDR-method provided one preserves the *b*_0_ component and keeps record of the removal current amplitudes.

A third limitation follows from the use of high-gain preamplifiers that have a reduced bandwidth and that either curtail the number of harmonics that can be demodulated or requires the use of lower excitation frequencies with accordingly longer spectroscopy times. High gain preamplifiers have not only a smaller bandwidth, they also require the application of several compensating voltages because the amplitudes of higher order current harmonics may still be too strong and saturate the preamplifier, as was partially the case in the topographic scan of Fig. 1(a). When more harmonics are removed, fewer demodulators are available for the measurement of information carrying resistive harmonics.

The noise that couples into the system via the compensation capacitor is also a limitation that needs attention since any signal transferred onto the current line will be amplified by the gain of the preamplifier. We observed the presence of noticeable power-supply related switching noise around 8 kHz when we changed the compensation capacitor from 1 pF to 10 pF. Likewise, harmonic distortion in the cosinusoidal compensation voltage might produce unwanted compensation currents. A potential remedy would be suitable low-pass filters, bandpass filters that only transmit the compensation frequency, and radiofrequency filters.

## CRediT authorship contribution statement

**Ajla Karić:** Validation, Investigation, Funding acquisition. **Carolina A Marques:** Investigation, Supervision, Funding acquisition. **Berk Zengin:** Investigation. **Fabian Donat Natterer:** Conceptualization, Methodology, Validation, Investigation, Writing – original draft, Supervision, Funding acquisition.

## Declaration of competing interest

The authors declare that they have no known competing financial interests or personal relationships that could have appeared to influence the work reported in this paper.

## Data Availability

Data will be made available on request.

## Footnotes

2 For even *n*: $cos^{n}\Theta =\frac{1}{2^{n}}\left(\begin{array}{c} n\\ \frac{n}{2} \end{array}\right)+\frac{2}{2^{n}}\sum_{k=0}^{\frac{n}{2}-1}\left(\begin{array}{c} n\\ k \end{array}\right)\cos\left((n-2k)\Theta \right)$ and for odd *n*: $cos^{n}\Theta =\frac{2}{2^{n}}\sum_{k=0}^{\frac{n-1}{2}}\left(\begin{array}{c} n\\ k \end{array}\right)\cos\left((n-2k)\Theta \right)$

3 Dynamic range =20log(50 nA/2.5 fA)=146 dB, where 2.5 fA represents the preamplifier noise level
